# Variations in KIR Genes: A Study in HIV-1 Serodiscordant Couples

**DOI:** 10.1155/2014/891402

**Published:** 2014-04-27

**Authors:** Vijay R. Chavan, Deepali Chaudhari, Swati Ahir, Zakiya Ansari, Preeti Mehta, Jayanti Mania-Pramanik

**Affiliations:** ^1^Department of Infectious Diseases Biology, National Institute for Research in Reproductive Health (DHR/ICMR), J. M. Street, Parel, Mumbai 400012, Maharashtra, India; ^2^Department of Microbiology, Seth G. S. Medical College & K.E.M Hospital, J. M. Street, Parel, Mumbai 400012, Maharashtra, India

## Abstract

*Background*. NK cells have anti-HIV activity mediated through killer cell immunoglobulin-like receptors (KIRs). The current prospective cohort study evaluated whether variation in KIR genes is associated with HIV infection in discordant couples (DCs), where one spouse remains seronegative (HSN) despite repeated exposure to the HIV. *Methods*. KIR was genotyped using PCR SSP. Viral load and CD4 counts were estimated using commercially available reagents. Data were analyzed using SPSS software. *Results*. Among the 47 DCs, HSN spouses had significantly (*P* = 0.006) higher frequencies of KIR3DS1. Regression analysis revealed significant (*P* = 0.009) association of KIR2DS1 with low viral load. KIR2DS4 variant was associated (*P* = 0.032) with high viral load. Three pairs of KIR genes were in strong LD in HSNs and two pairs in HSPs. There were 60 KIR genotypes, and 16 are reported the first time in the Indian population. Exclusive genotypes were present either in HSPs (*N* = 22, 11 unique genotypes) or in HSNs (*n* = 27, 9 unique genotypes). *Conclusions*. This study highlights for the first time in the Indian population an association of KIR genes in HIV infection where presence of exclusive and unique genotypes indicates possible association with either HIV infection or with protection.

## 1. Introduction

Variations in genetic and immunological factors are associated with protection from HIV infection in HIV exposed seronegative individuals [1]. Natural killer (NK) cells, one of the critical components of the innate immune system, have great potential to mediate antiviral activity at the earliest stage of HIV infection. Functional analysis of NK cells, during HIV infection, suggests a potential role in controlling virus proliferation [2, 3]. In this respect NK cells have the capacity to kill tumor or virus infected cells without prior sensitization, leading to an effective antiviral immune response [4, 5]. Though there are many receptor ligands on human NK cells, they largely use Killer cell immunoglobulin-like receptors (KIRs) to distinguish diseased targets from healthy cells [6]. However, immunogenetic analysis of different populations shows significant differences in terms of KIRs gene frequency and haplotype distribution [7, 8]. Hence, it is important to understand KIR gene distribution in any population to assess their potential association with HIV infection and disease progression.

The KIR locus spans a region of about 150–200 kb on chromosome 19q13.4 and is part of the leukocyte receptor complex (LRC) [9]. There are 15 KIR genes and two pseudogenes. Twelve of these genes encode for receptors with two immunoglobulin domains (KIR2D genes) while 5 genes encode for receptors with three immunoglobulin domains (KIR3D genes) (http://www.ebi.ac.uk/ipd/kir:). A KIR can inhibit or activate NK-cell function depending on the type of signaling it transduced through its cytoplasmic tail. The KIR haplotypes can be split into two basic groups based on their gene content; groups A and B. The group A haplotype contains one activating KIR gene KIR2DS4 and six inhibitory KIR genes: KIR2DL1, KIR2DL3, KIR2DL4, KIR3DL1, KIR3DL2, and KIR3DL3. Group B haplotypes are more diverse as they have more activating receptors and are characterized by 2DL2, 2DS1, 2DS2, 2DS3, and 2DS5 genes [7, 8].

NK cell function and KIR have not only been linked to HIV disease progression, but also with resistance to HIV infection in certain populations of exposed uninfected individuals [10–12]. No report from India is available on association of KIRs with HIV infection. India being home to the world's third largest population with HIV/AIDS (http://www.unaids.org:) gives an opportunity to study such association of KIRs with HIV infection. Study of HIV discordant couples (DCs), where one spouse is HIV seropositive (HSP) while the other remains HIV seronegative (HSN) in spite of repeated exposure, offers more scope to identify potential “immunologic advantages” that characterize HSNs. Hence, the present study was undertaken to evaluate the variation in KIR genes in HIV-1 discordant couples to assess their possible association with HIV infection within the Indian population.

## 2. Methods

### 2.1. Study Participants

The prospective cohort study on HIV-1 DCs was conducted during 2010–2013 with approval of the Institutional and King Edward Memorial (KEM) Hospital Ethics Committees. Individuals or couples attending the Integrated Counseling and Testing Centre (ICTC), of Department of Microbiology of Seth G.S. Medical College and KEM Hospital, Mumbai, India, for HIV screening either voluntarily or as a reference case were informed about the study. Eligible couples were identified as DCs, where one partner was HSP while the other had unprotected sexual relationship but was diagnosed as HIV seronegative (HSN). Detail history of the DCs such as year of marriage, time spent together/separate, positive spouse HIV detection date, route of transmission, ART status, opportunistic infections, and 1st screening date of HSN spouse was recorded. The clinical information was collected prospectively by well-trained hospital counselors who individually counseled each participant before and after HIV testing. Those who turned out to be positive for HIV-1 infection were counseled to bring his/her spouse for HIV testing. Based on the willingness of the spouse for participation in HIV testing and follow-up, the couples were enrolled in the study. The prevalence rate of DCs among HIV infected individuals residing at Mumbai was not available to calculate the sample size. Sample size was dependent on the number of available DCs, who completed more than 2 years of follow-up [13]. A written informed consent document was taken from all the couples before their enrollment in the study.

### 2.2. HIV Screening

Blood samples (5 mL) were collected in two vials, one in EDTA and another in plain vacutainer (BD Biosciences) from every HIV-1 DC enrolled in this study. Serum was tested for HIV antibodies by three rapid tests of different antigens/principles (testing strategy III) as per the guidelines of National AIDS Control Organization (NACO), Government of India (http://nacoonline.org:). The HIV status of each HSN spouse was further confirmed by HIV DNA PCR test [14].

### 2.3. DNA Extraction

DNA was extracted from whole blood using a salting out procedure with minor modifications [15]. This was used for HIV DNA PCR and KIR genotyping.

### 2.4. Follow Up of Couples

Both HSPs and HSNs were followed up for as long as they were willing to attend following the date of screening of HIV status. Follow-up date was written on the clinical file of the HSP spouse. If they consented for a telephone reminder, they were contacted by the counselors. The HSP spouses were followed up to register their health status by CD4 count, disease management, and for free treatment. The HSN spouses were counseled for follow-up to confirm their HIV status. Free condoms were provided throughout the study. The HSN spouses were followed up based on their current living relationship status. During the follow-up if the HSN spouse turned out to be positive, the couple was excluded from the analysis.

### 2.5. Estimation of CD4 Count

CD4 count was done using BD FACS count CD4 reagents from BD Biosciences (Bectson Dickinson and Company, San Jose, CA) following the instruction manual provided by the manufacturer. Appropriate amount of monoclonal antibody reagent and whole blood (50 *μ*L) was directly added to the tube to dissolve the lyophilized pellet, releasing a known number of fluorescent beads. During analysis, the absolute number (cells/*μ*L) of positive cells in the sample was determined by comparing cellular events to bead events using BD Multi SET software (supplied with the instrument, BD FACSCalibur).

### 2.6. Determination of Viral Load

The viral load analysis was done in plasma with the isolation of total nucleic acid using the MagNa pure Compact (Roche Diagnostic, Germany). Subsequently, the viral load was measured by Cobas Taqman Real time PCR (Roche Molecular Systems, USA) according to the manufacturer's instructions. The lower detection limit was 34 RNA copies of virus per mL of the plasma. Estimated viral load was divided into three groups (low, medium, and high). To assess the quality of the viral load results, randomly selected samples such as those with undetectable, intermediate, and with high viral load values were reassessed.

### 2.7. KIR Genotyping

KIR genotyping was done using PCR-SSP (Inno-TRAIN, Diagnostic GmbH, Germany) according to the manufacturer's instructions. A total of 23 pairs of specific primers were used to identify the presence or absence of 14 KIR genes (KIR2DL1, 2DL2, 2DL3, 2DL4, 2DL5, 2DS1, 2DS2, 2DS3, 2DS4, 2DS5, 3DL1, 3DL2, 3DL3, and 3DS1) and two pseudogenes (2DP1 and 3DP1). The KIR2DS4 gene based on a 22 bp deletion in exon 5 region of the gene was further subtyped as KIR2DS4 normal and KIR2DS4 deleted (variant). All the amplified samples were run on 2% agarose gel and subjected to electrophoresis, where specific amplificates separate in the electrical field according to size. The band pattern of KIR specific amplicons and internal controls (HGH 430 bp and 800 bp) was visualized by intercalating ethidium bromide dye and detected under the UV light. In the case of unique genotypes (genotypes that were not reported earlier), genotyping of those samples was repeated.

### 2.8. KIR Haplotypes and Genotypes

Individuals with KIR 2DL1, 2DL3, 3DL1, and 2DS4 genes were grouped under A haplotype, while individuals with KIR 2DL2, 2DL5, 2DS1, 2DS2, 2DS3 2DS5, and 3DS1 genes were grouped under B haplotype. If an individual did not have a single gene of group B haplotype, it was considered as having AA genotype. If all the B haplotype genes were present and group A haplotype genes were absent, then they were grouped under BB genotype [16]. AB genotypes were considered as heterozygous carrying both haplo group genes. However, the current system did not distinguish between AB and BB genotypes and therefore collectively were annotated as Bx (http://www.allelefrequencies.net:).

### 2.9. KIR GIDs

The allele frequency net database (AFND) available at http://www.allelefrequencies.net is an online repository that contains information on the frequencies of immune genes and their corresponding alleles in different populations of the world. The KIR genotypes consist of 16 KIR genes, of which some may be present or absent in a specific genotype combination. In this system, the genotype combination (GIDs) for each individual was searched using a freely available interactive online database at the website. In this data base, there are 492 different KIR genotypes reported in 16,988 individuals from 143 populations of the world. An individual with a specific genotype (AA/Bx) is assigned with a specific GID (Example: 1, 195, etc.) available in this data base. However, if this identified genotype is not in the database, it is considered as a unique or novel genotype.

### 2.10. Linkage Disequilibrium (LD) Analysis

Linkage disequilibrium (LD) is a measure of association of nonrandom alleles at two different loci. LD was used in this study to assess the genetic association between KIR gene pairs. The LD analysis was done using Haploview (version 4.2) software. In this the strength of the association between the two genes is dependent on *D*′ (linkage disequilibrium) and *r* ^2^ (relative linkage) value.

### 2.11. Statistical Analysis

Data analysis was done using SPSS software (version 19.0, SPSS, USA). Comparison of the KIR gene frequencies of HSPs with HSNs was performed using 2 × 2 contingency tables (Stat calc program, Epi Info v. 6.04, USA). A *P* value < 0.05 was considered significant. Bonferroni correction was further applied to determine significance level (0.05/*n* where “*n*” is the sum of conditions in whole study). Comparison between the mean average age in HSP and HSN spouses was done using Mann-Whitney *U* test. Association of KIR genes with log transformed viral load and median CD4 counts as dependent variables was done using linear regression models, where strength of the association is defined by the *β* value.

## 3. Results

### 3.1. Subjects Demographic Details

**Figure 1:**
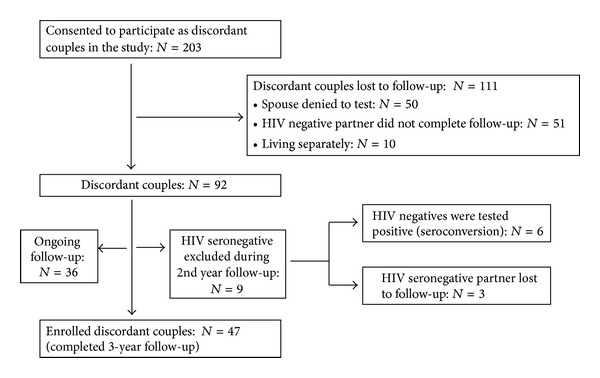
Enrollment of discordant couples.

Forty-seven HIV-1 DCs (staying together and having unprotected sexual relationships) were enrolled in this study (Figure 1). All the DCs included in this study were aware of their partner's HIV status. The detailed demographic and clinical characteristics of discordant couples are presented in Table 1. All the discordant couples were living together for a period of 1 to 28 years even after detection of HIV in the HSP spouse. The time interval between the HIV detection in one spouse and screening of the other spouse varied from 0 (immediate) to 6 years. Immediate HIV screening of the HSN spouse was carried out in 17 couples. Sexual behavior histories of the couples were collected by well-trained hospital counselors during follow-up for each spouse of the DCs. While collecting the sexual behavior history counselors interviewed each of the spouses separately and did not disclose any history reported by the individuals to their corresponding spouses.

Even after counselling the DCs to use condoms, thirty-two spouses revealed having unprotected relationships (4-5 times or more/month) with their spouses within 6 months of their enrollment. About 15 discordant couples did use a protective method during sexual contact but there was lack of consistent condom use. The frequency of coitus, that is, vaginal intercourse between these couples varied from 1 to 3 per month. The number of follow-ups of the HSNs was at least 2 times/year for a minimum period of 2 years.

There was no significant difference (Mann Whitney *U* test *P* = 0.06) between the mean average age of HSPs (36.2 ± 8.8 years) and HSNs (33.6 ± 6.5 years). The median CD4 count for HSPs was 303 ± 201 cells/*μ*L, and 17% of them had CD4 count <200 cells/mm^3^. The median viral load of HSPs was found to be 22137RNA copies/mL (4.34 Log_10_RNAcopies/mL, range: 34–1182648). Randomly selected samples (*n* = 16), such as those with undetectable values (*n* = 5), high viral load values (*n* = 4) and those with intermediate values (*n* = 7) were reassessed during quality assessment and the results were reproducible. None of the HIV seropositive (HSP) spouses were on ART before enrollment in the study. However during the three years of follow-up 38.3% of the HSPs were initiated ART regimen and 61.7% were not on ART, this difference was statistically significant (*P* = 0.04). Among the HSPs, 29.8% had opportunistic infections (OIs) and/or coinfection (CIs).

### 3.2. KIR Gene Frequencies

Frequencies of individual KIR genes in DCs are presented in Table 2. Frequencies of different KIR genes varied from 27.6% to 97.8%. Activating gene frequency was 51.0% to 91.4%. Frequency of inhibitory KIR genes varied from 51.0% to 97.8%. Pseudogene frequency was 27.6% to 93.6%. HSNs had significantly higher frequencies of activating KIR3DS1gene compared to HSPs (91.4% versus 72.3%, *P* = 0.006), which remained significant even after application of the Bonferroni corrections. HSPs had significantly higher frequencies of inhibitory gene KIR2DL5 compared to HSNs (74.6% versus 51.0%, *P* = 0.03); however, this was nonsignificant after Bonferroni correction. Frequencies of all other genes were almost similar in both HSPs and HSNs. There was also no variation in distribution of pseudogenes in both the groups. All three framework genes (KIR3DL2; -3DL3; -2DL4) were present in 85.1% (80 of 94) of the studied population. In the rest either one or two of these frame work genes were present.

### 3.3. KIR Genotypes and Haplotypes

**Figure 2:**
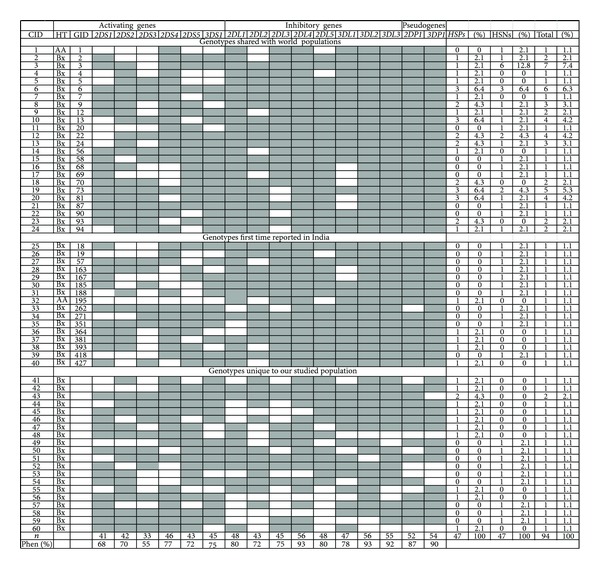
KIR genotyping in HSP and HSN spouses. Filled boxes with grey color represent presence of gene and white boxes represent absence of gene. CID: combination id; HT: haplotype; GID: genotype id; HSP: HIV-1 seropositive; HSN: HIV seronegative uninfected; %: percentage of individuals; Bx: genotype (AB + BB); *n*: the total number of positives in genotypes; Phne%: phenotype percentage.

Genotype and haplotype analysis revealed that only two individuals had A haplotypes and AA genotype (HSP: GID195 and HSN: GID 1). All others were with B haplotype (*n* = 92) and were annotated as Bx genotype. Genotyping of the studied population revealed 60 genotypes and their frequency varied from 1.1% to 7.4%. Forty genotypes were shared with the genotypes of World populations, reported in the allele frequencies net database. Among these, 16 genotypes were reported for the first time in the Indian population. Another 20 genotypes were unique and not reported in any populations of the world. Among the genotypes shared with other population, GID 3 had a frequency of 7.4%. This GID frequency was higher amongst HSNs compared to HSPs (12.8% versus 2.1%). However this difference was not statistically significant. There was no difference in frequency of genotypes between HSPs and HSNs. Phenotypic frequency of these genes varied from 55.0% to 93.0% among the studied population (Figure 2).

### 3.4. Distribution of KIR3DS1/L1 in HSP and HSN Spouses

KIR3DS1 homozygote (3DS1hmz), KIR3DS1/L1 heterozygotes (htz), and KIR3DL1 homozygote (3DL1hmz) data is presented in Table 3. This data shows that KIR3DS1/L1 heterozygotes were significantly higher in HSNs as compared with HSPs (*P* = 0.03).

### 3.5. Genotype Combinations (GIDs) Exclusively Present in Either HSPs Or HSNs

There were some genotypes present exclusively in HSPs and HSNs (Table 4, Figure 2). Of these exclusive genotypes, some genotypes were unique, previously not reported in the database.

### 3.6. Comparative Analysis of KIR Genes with Viral Load and CD4 Counts

Comparative analysis between KIR genes with viral load and CD4 counts of HSPs by linear regression model revealed significant association of activating gene KIR2DS1 with viral load control in HSP, while KIR2DS4-deleted variant was associated with high viral load (Table 5). There was significant association of KIR2DS1 with low viral load and KIR2DS4-deleted variant with high viral load when compared across 3 VL categories (Table 6). The median viral load in 15% of HSPs who did not have the KIR2DS4-normal or deleted variant was 2.93 log_10_ RNA copies/mL.

### 3.7. Linkage Disequilibrium Analysis

**Figure 3:**
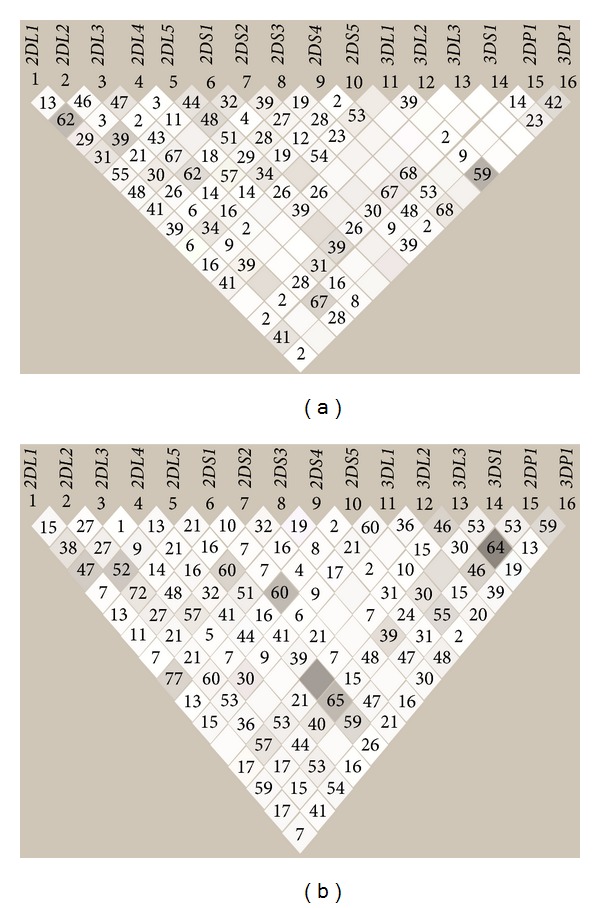
Linkage disequilibrium analysis between HSN (a) and HSP (b) haplotype structure for the 16 KIR genes HSN (a) and HSP (b) spouses. The top panel shows *R*-squared color scheme with LD values the two groups used in the case-control analysis. *r* ^2^ Values are represented white for *r* ^2^ = 0, with intermediate values for 0 < *r* ^2^ < 1 indicated by shades of grey. The numbers within the squares represent the *r* ^2^ scores for pairwise LD.

There were five pairs of KIR genes that showed significant linkage disequilibrium (LD) in HSPs and HSNs (Figure 3). In HSNs, three pairs of KIR genes were in strong LD (KIR 2DS5-3DL1, 2DS1-3DL1, and 2DS1-3DP1). For HSPs, two pairs of KIR genes were in strong LD (2DL5-3DL3, 3DL1-3DS1) (Table 7).

## 4. Discussion

Our study of variation within KIR genes in HIV discordant couples highlights a significant increase in frequency of the activating genes KIR3DS1 and KIR3DS1/L1 heterozygotes in HSNs. Activating gene KIR2DS1 was associated significantly with low viral load in HSPs, while a 22 bp deletion in the exon 5 of KIR2DS4 gene (variant) was associated with high viral load. Among the 47 discordant couples, we identified sixty genotypes, of which 40 are shared with global genotypes. Sixteen of these 40 genotypes are reported for the first time in the Indian population. We also identified 20 unique genotypes, not reported previously. Identification of exclusive and unique genotypes either in HSPs or HSNs suggests their possible association either with susceptibility or protection to infection. LD analysis shows that in HSPs two pairs of genes are in strong linkage. While in HSNs three pairs of genes are strongly linked emphasizing that they may have some protective effect.

Studies from different parts of the world suggest that HIV negative partners in HIV discordant relationships are at increased risk of acquiring HIV infection. A substantial number of new HIV infections occur within stable relationships [17–19]. In India, heterosexual contact is the major route of infection (http://nacoonline.org:). Women are more likely to be the HIV uninfected partner in discordant relationships [19]. However, in our study a high number (36.1%) of women were HIV-1 positive in discordant relationships. The median viral load in HSPs was found to be 22137 RNA copies/mL; however, this was lower than an earlier study on Western Indian discordant couples [20]. Initiation of ART for HSPs in discordant relationships has decreased the rate of HIV transmission [21]. In our study 38.3% of the HSPs were enrolled into ART after their participation. The 61.7% of HSNs who's partners were not on ART were at greater risk; however, during the minimum 2-year follow-up they remained uninfected.

Opportunistic infections or coinfections (OIs/CIs) are cofactors of HIV transmission [22]. The current study revealed a low frequency of OIs/CIs (34%) among the HSPs. This might be associated with reduced HIV transmission to their HSN spouses.

Analysis of KIR genes in 47 HIV-1 DCs revealed a high frequency of KIR2DL5 among the HSPs. Significantly higher frequencies of inhibitory genes KIR2DL2 and KIR2DL5 were previously observed in exposed uninfected African female sex workers (EUFSW) [11]. This discrepancy emphasizes further studies to confirm the association of these inhibitory receptors with transmission in different populations. We have observed a significantly higher frequency of the activating gene KIR3DS1 in HSNs. This observation is similar to an earlier study on HIV exposed uninfected Canadian population [12]. Conversely, we have observed significantly higher frequencies of KIR3DS1/L1 heterozygotes in HSNs while they have reported KIR3DS1 homozygotes in HIV exposed uninfected individuals [12]. A recent study of a Chinese's population reported an increased proportion of KIR3DS1/L1 heterozygotes in long term nonprogressors (LTNPs), associated with slower CD4 cell decline, indicating a protective effect [23]. The presence of KIR3DS1 leads to increased IFN-gamma expression compared with individuals lacking KIR3DS1 [24]. A study of HIV-1 infected European populations has shown that, regardless of KIR3DL1 status, an increase in the effective count of KIR3DS1 has a protective effect. NK cells from individuals with multiple copies of KIR3DL1, in the presence of KIR3DS1, and the appropriate ligands inhibit HIV-1 replication. However, NK cells derived from individuals with multiple copies of effective KIR3DS1 in the absence of an effective KIR3DL1 did not appear to mediate robust antiviral activity in vitro [25]. These studies support the possible association of KIR3DS1/L1 heterozygotes with protection.

The other significant observation in this study was the association of KIR2DS1, an activating receptor gene, with low viral load in HSPs. This confirms earlier findings in a South African population where an increase in KIR2DS1 genes was associated with decreasing HIV-1 viral load [26]. In our study HSPs with the KIR2DS4-deleted variant had high viral loads. The KIR2DS4-deleted variant differs from the normal KIR2DS4 gene sequence by a 22 bp deletion in exon 5, which causes a frame shift, yielding a truncated KIR2DS4 protein with loss of the transmembrane and cytoplasmic domains of the full-length KIR2DS4 protein [27]. Since both of these types are quite common in the global population, they are analyzed separately. Both KIR2DS4-normal and its' deleted variant were absent in 15% of HSPs, higher than an earlier report (3.2%) on Zambian discordant couples [28]. This earlier study indicated that functional KIR2DS4-normal was associated with relatively high viral load; however, we observed significant association of the KIR2DS4-deleted variant with high viral load.

We did not find any significant association between KIR genes and CD4 counts.

KIR genotype/GID analysis revealed two individuals with an AA genotype, one of them (GID: 195) is reported for the first time in the Indian population. All the other study individuals had a Bx (AB + BB) genotype, similar to previous reports for the Indian populations [29]. Identification of additional unique genotypes in the studied population adds to the existing knowledge of KIR genotypes. These unique along with other specific GIDs, which were exclusively present either in the HSPs or in HSN spouses, highlight their association with either acquisition or with protection from HIV infection.

The KIR genes are tightly packed within a 200 kb region. The organization of the KIR locus in a head-to-tail fashion probably facilitates gene expansion by duplication and recombination and is therefore reflected by the linkage disequilibrium [30]. A previous study has reported strong LD between KIR3DS1 and KIR2DS1 and KIR3DL1 with KIR3DS1 [31]. In HSPs, we found specific LD between KIR3DL1 with KIR3DS1, besides a LD between KIR2DL5 and KIR3DL3. However, in HSN, KIR3DL1 was strongly linked with KIR2DS1 and KIR2DS5, while KIR2DS1 was linked with Pseudogene KIR3DP1. These observed linkages between inhibitory and with activating genes may have specific association either with HSP or HSNs.

Small sample size was the major study limitation, reflecting difficulties in establishing a study cohort of serodiscordant couples in Indian populations due to confidentiality, discrimination, and social stigma. We have successfully developed this cohort with 3 years of follow-up. Due to financial constraints, we were unable to perform HLA analysis to support the association of specific KIR-HLA role in HIV infection. However, to the best of our knowledge ours is the first and only study highlighting the potential association of KIR genes with HIV infection from India.

## 5. Conclusions

In conclusion, our study highlights the presence of specific activating genes associated either with protection or viral load. Linkage of an inhibitory gene with one or more of activating genes in HSNs is possibly associated with protection. Sixteen KIR genotypes were identified for the first time in the Indian population. The presence of 20 unique as well as exclusive genotypes indicated their possible association either with HIV infection or with protection.

## Figures and Tables

**Table 1:** Demographic and clinical characteristics of enrolled HIV-1 discordant couples (*n* = 47).

Variables	
Cohort period	2010–2013
Number of discordant couples enrolled	47
Male:female ratio	30 : 17
Age of seropositives (HSPs) (mean ± SD)	36.2 ± 8.8 yrs
Males (*n* = 30)	41.03 ± 5.35 yrs
Females (*n* = 17)	27.8 ± 7.2 yrs
Age of exposed seronegatives (HSNs)	33.6 ± 6.5 yrs
Males (*n* = 17)	32.7 ± 8.2 yrs
Females (*n* = 30)	34.2 ± 5.3 yrs
Opportunistic/coinfections	12 (25.5%) TB (M: 10; F: 2)
	3 (6.03%) Herpes zoster (M: 1; F: 2)
	2 (4.2%) Syphilis (M: 2)
Route of HIV transmission	
Heterosexual	28 (59.6%)
Blood transfusion	3 (6.4%)
Infected needle prick	3 (6.4%)
Not known	13 (27.7%)
ART given after enrollment	18 (38.3%) (M: 11; F: 7)
Not on ART	29 (61.7%) (M: 19; F: 10)
Duration of exposure before enrollment (mean ± SD)	16.5 ± 11.2 months
Duration of exposure after enrollment (mean ± SD)	12.3 ± 9.4 months
Median HIV-1 viral load (log_10_⁡)	22137 RNA copies/mL (4.34 log_10_⁡)
	UD: 6, <1000: 11, 1001–9999: 10, 10,000–50,000: 8, 50,001–1,10,000: 2, >1,10,000: 10
UD viral load in HSPs after ART initiation	*n* = 4
CD4 count (median ± SD)	303 ± 201 cells/*μ*L
HSPs with <200 CD4 count	8 (17%)

TB: tuberculosis, ART: antiretroviral therapy, RNA: ribonucleic acid, M: male, F: female, UD: undetectable.

**Table 2:** Activating/inhibitory KIR gene repertoire in HSP (*N* = 47) and HSN (*N* = 47) individuals.

	HSPs *N* = 47 (%)	HSNs *N* = 47 (%)	OR	95% CI	*P*	*P*-adj
Activating genes						
2DS1	30 (63.8)	32 (68.0)	0.82	0.35–1.94	0.828	0.035
2DS2	40 (85.1)	34 (72.3)	0.89	0.38–2.11	0.207	0.014
2DS3	30 (63.8)	24 (51.0)	1.69	0.74–3.86	0.297	0.021
2DS4-Nor	40 (85.1)	42 (89.0)	0.68	0.16–2.65	0.759	0.028
2DS4-Del	38 (80.8)	38 (80.8)	1.00	0.36–2.89	1.000	0.042
2DS5	35 (74.4)	36 (76.5)	0.89	0.34–2.28	1.000	0.050
3DS1	34 (72.3)	43 (91.4)	0.24	0.07–0.81	0.006*	0.007^∧^
Inhibitory genes						
2DL1	38 (80.8)	40 (85.1)	0.73	0.25–2.18	0.411	0.011
2DL2	37 (78.2)	38 (80.1)	0.87	0.31–2.40	1.000	0.044
2DL3	34 (72.3)	36 (76.5)	0.79	0.31–2.02	0.813	0.038
2DL4	40 (85.1)	42 (89.3)	0.68	0.19–2.31	0.759	0.027
2DL5 A	28 (59.5)	32 (68.0)	0.69	0.29–1.60	0.520	0.016
2DL5 B	35 (74.6)	24 (51.0)	2.79	1.17–6.67	0.032*	0.005
3DL1	37 (78.7)	39 (82.9)	0.75	0.27–2.13	0.793	0.033
3DL2	45 (95.7)	45 (95.7)	1.00	0.13–7.41	1.000	0.050
3DL3	44 (93.6)	46 (97.8)	0.31	0.31–3.18	0.617	0.022
Pseudogenes						
2DP1	44 (93.6)	43 (91.4)	1.36	0.28–6.46	1.000	0.050
3DP1*003	39 (82.9)	41 (87.2)	0.71	0.22–2.24	0.773	0.033
3DP1*001-004	13 (27.6)	17 (36.1)	0.67	0.28–1.61	0.507	0.016

HSPs: HIV seropositive spouses; HSNs: HIV seronegative spouses; OR: odds ratio; CI: confidence interval; *P-values <0.05 are considered statistically significant; *P-*adj: bonferroni correction values; Nor: normal; Del: deleted. ^∧^Considered significant after boneferroni correction.

**Table 3:** Distribution of KI3DS1/L1 in HSP and HSN spouses (*n* = 47).

KIR 3DS1	HSPs	HSNs	OR	95% CI	*P*
3DS1/3DS1 (hmz)	10 (21.3%)	7 (14.9%)	1.544	0.476–5.092	0.593
3DS1/3DL1 (hetz)	24 (51.0%)	35 (74.5%)	0.358	0.136–0.928	**0.032**
3DL1/3DL1 (hmz)	13 (27.7%)	5 (10.6%)	3.212	0.937–11.590	0.065

HSPs: HIV seropositive spouses; HSN: HIV seronegative spouses; hmz: homozygotes; hetz: heterozygotes; OR: odds ratio; CI: confidence interval; *P*: <0.05.

**Table 4:** GIDs exclusively present either in HSPs or HSNs.

	HSPs (*n* = 22)	HSNs (*n* = 27)
GIDs number	4,5,7,56,70,93,195,364,381,393,427	1,13,18,19,57,58,68,69,87,90,163,167,185,188,262,271,351,418,
	Unique genotypes: 11	Unique genotypes: 9

HSPs: HIV seropositive spouses, HSNs: HIV seronegative spouses, GIDs: genotype id.

**Table 5:** KIR genes association with viral load and CD4 count in HSPs by linear regression (*n* = 47).

KIR	Frequency (*N* = 47) %	HIV-1 viral load (log_10_⁡)	*β*	*P*	Median CD4+ T cell count (cells/*µ*L)	*β*	*P*
2DL1	80.8	3.91	−0.170	0.287	322	0.251	0.873
2DL2	78.2	3.94	−0.250	0.115	304	0.218	0.161
2DL3	72.3	3.86	−0.088	0.582	331	−0.074	0.638
2DL4	85.1	3.76	0.027	0.865	328	−0.056	0.721
2DL5 A	59.5	3.48	0.227	0.153	304	0.126	0.420
2DL5 B	74.6	3.63	0.159	0.321	317	0.066	0.672
2DS1	63.8	3.27	0.404	0.009*	341	−0.127	0.417
2DS2	85.1	3.79	−0.034	0.835	318	0.072	0.647
2DS3	63.8	3.74	0.034	0.835	340	−0.174	0.266
2DS4-Nor	85.1	3.55	0.163	0.307	314	−0.055	0.726
2DS4-Del	80.8	3.99	−0.336	0.032*	295	−0.196	0.207
2DS5	74.4	3.59	0.188	0.238	322	0.018	0.911
3DL1	78.7	3.99	−0.287	0.069	342	−0.185	0.235
3DL2	95.7	3.82	−0.184	0.250	334	−0.258	0.095
3DL3	93.6	3.79	−0.074	0.644	322	0.037	0.812
3DS1	72.3	3.58	0.218	0.172	327	−0.033	0.835
2DP1	93.6	3.74	0.086	0.595	324	−0.011	0.943
3DP1*003	82.9	3.70	0.105	0.512	333	0.116	0.458
3DP1*001-004	27.6	3.64	0.060	0.707	299	0.082	0.602

HSPs: HIV seropositive spouses; OR: odds ratio; CI: confidence interval; *P values <0.05 are considered statistically significant; *β*: beta value.

**Table 6:** Association of KIR2DS1 and KIR2DS4-del variant with HIV-1 viral load in HSPs.

KIR gene	HSPs *N* (%)				
	Viral load			Linear regression values	
	Low (<5000 copies/mL)	Medium (5000–10,000 copies/mL)	High (>10,000 copies/mL)	*β*	*P*
KIR2DS1+	15 (50.0%)	8 (26.7%)	7 (23.3%)	0.688	0.000*
KIR2DS1−	1 (6.9%)	2 (11.7%)	14 (82.3%)	0.644	0.007*
KIR2DS4-del+	14 (36.8%)	3 (7.9%)	21 (55.3%)	0.871	0.000*
KIR2DS4-del−	3 (33.3%)	—	6 (66.7%)	0.571	0.181

HSPs: HIV-1 seropositive spouses; KIR: killer immunoglobulin receptor; %: percentage; del: deleted; +: gene present; −: gene absent; *P values <0.05 are considered statistically significant; *β*: beta value.

**Table 7:** Linkage-disequilibrium (LD) analysis of 16 KIR genes between HSPs and HSNs.

	2DL1	2DL2	2DL3	2DL4	2DL5	2DS1	2DS2	2DS3	2DS4	2DS5	3DL1	3DL2	3DL3	3DS1	2DP1	3DP1
2DL1	*D*′ *r* ^2^	0.153 0.021	0.386 0.092	0.47 0.163	0.072 0.005	0.13 0.007	0.117 0.01	0.078 0.001	0.773 0.136	0.13 0.001	0.153 0.021	1.0 0.011	0.175 0.009	0.598 0.032	0.175 0.009	0.072 0.005

2DL2	0.139 0.017		0.277 0.008	0.274 0.049	0.524 0.208	0.724 0.08	0.274 0.049	0.217 0.022	0.217 0.013	0.608 0.034	0.53 0.021	0.365 0.022	0.577 0.084	0.171 0.021	0.153 0.006	0.412 0.009

2DL3	0.627 0.225	0.466 0.014		0.013 0.0	0.096 0.001	0.149 0.005	0.484 0.016	0.575 0.072	0.057 0.001	0.078 0.006	0.309 0.067	1.0 0.017	0.539 0.052	0.444 0.029	0.539 0.052	0.548 0.024

2DL4	0.295 0.059	0.036 0.001	0.478 0.089		0.139 0.017	0.21 0.004	0.161 0.026	0.329 0.033	0.416 0.029	0.44 0.012	0.093 0.006	1.0 0.008	0.217 0.018	0.408 0.076	1.0 0.012	0.161 0.001

2DL5	0.311 0.083	0.397 0.158	0.021 0.0	0.036 0.001		0.217 0.017	0.161 0.001	0.608 0.134	0.51 0.056	0.161 0.015	0.412 0.009	0.397 0.034	**1.0** **0.332**	0.654 0.23	0.598 0.119	0.266 0.003

2DS1	0.552 0.025	0.217 0.005	0.43 0.027	0.119 0.004	0.449 0.088		0.105 0.003	0.078 0.006	0.078 0.004	0.608 0.224	0.06 0.002	0.217 0.004	0.078 0.0	0.156 0.017	0.478 0.027	0.217 0.017

2DS2	0.484 0.016	0.309 0.051	0.671 0.053	1.0 0.046	0.482 0.124	0.322 0.085		0.329 0.033	0.161 0.005	0.041 0.001	0.093 0.006	1.0 0.008	1.0 0.012	0.484 0.016	1.0 0.012	0.161 0.001

2DS3	0.416 0.029	0.266 0.015	0.628 0.116	0.183 0.004	0.51 0.056	0.046 0.001	0.397 0.063		0.194 0.022	0.086 0.004	0.171 0.004	1.0 0.025	0.078 0.0	0.397 0.107	0.478 0.027	0.309 0.011

2DS4	0.39 0.023	0.06 0.001	0.145 0.007	0.573 0.034	0.295 0.02	0.288 0.034	0.277 0.033	0.199 0.036		0.021 0.0	0.217 0.013	0.021 0.0	0.247 0.024	0.319 0.007	0.489 0.047	0.608 0.034

2DS5	0.067 0.003	0.347 0.081	0.169 0.029	0.145 0.001	0.347 0.081	0.199 0.026	0.12 0.012	0.288 0.026	0.029 0.0		0.608 0.034	1.0 0.015	0.105 0.002	0.309 0.086	0.552 0.061	0.021 0.0

3DL1	0.161 0.001	0.096 0.009	0.021 0.0	1.0 0.024	0.266 0.003	**1.0** **0.096**	0.548 0.024	0.234 0.011	0.53 0.065	**1.0** **0.063**		0.365 0.022	0.153 0.006	**1.0** **0.103**	0.153 0.006	0.206 0.032

3DL2	0.413 0.043	0.397 0.034	1.0 0.014	1.0 0.005	0.397 0.034	0.266 0.007	1.0 0.017	1.0 0.043	1.0 0.039	1.0 0.014	0.397 0.034		0.466 0.141	0.309 0.011	0.466 0.141	0.397 0.034

3DL3	1.0 0.004	1.0 0.106	1.0 0.007	1.0 0.003	1.0 0.006	1.0 0.046	1.0 0.057	1.0 0.023	1.0 0.025	1.0 0.007	1.0 0.004	1.0 0.001		0.539 0.052	0.644 0.415	0.197 0.013

3DS1	0.02 0.0	0.021 0.0	0.288 0.004	0.312 0.079	0.397 0.113	0.266 0.022	0.309 0.037	0.674 0.069	0.687 0.078	0.13 0.008	0.021 0.0	1.0 0.007	1.0 0.003		0.539 0.052	0.136 0.01

2DP1	0.412 0.09	1.0 0.019	0.674 0.138	0.161 0.02	1.0 0.019	1.0 0.044	0.096 0.0	0.489 0.021	0.53 0.03	1.0 0.028	0.096 0.004	1.0 0.004	1.0 0.002	0.14 0.013		0.598 0.119

3DP1	0.021 0.0	1.0 0.03	0.288 0.004	0.083 0.006	1.0 0.03	**1.0** **0.069**	0.397 0.009	0.021 0.0	0.687 0.078	1.0 0.045	0.598 0.255	1.0 0.007	1.0 0.003	0.236 0.056	0.427 0.116	

The upper triangle represents HSPs and the lower triangle represents HSNs. HSPs: HIV seropositive spouses; HSNs: HIV seronegative spouses; LD plot with first line *D*′: linkage disequilibrium value; second line *r* ^2^: relative linkage disequilibrium value; bold numbers representing genes in significant LD.
